# Little association of biological trait values with environmental variables in invasive alien round goby (*Neogobius melanostomus*)

**DOI:** 10.1002/ece3.2942

**Published:** 2017-04-26

**Authors:** Alexander F. Cerwenka, Alfredo Pagnotta, Carolin Böker, Joerg Brandner, Juergen Geist, Ulrich K. Schliewen

**Affiliations:** ^1^ Aquatic Systems Biology UnitTechnical University of MunichFreisingGermany; ^2^ SNSB‐Bavarian State Collection of Zoology (ZSM)MünchenGermany; ^3^ Wasserwirtschaftsamt RegensburgRegensburgGermany

**Keywords:** aquatic invasive alien species, biological trait variation, individual trait utility hypothesis, local environmental factors, *Neogobius melanostomus*

## Abstract

The relative importance of species‐specific biological trait characteristics and environmental factors in invasions of nonindigenous species remains controversial because both have mostly been studied independently. Thus, the main objective of this study was to examine the correlation of biological traits with environmental variation in the globally invasive round goby *Neogobius melanostomus* from the upper Danube River. Based on a sample of 653 specimens along a continuous 200 km river pathway, links between nine environmental factors (substrate‐type, six water measurements, and the communities of fishes and macroinvertebrates) and seven biological traits (nutritional and energetic status, trade‐offs of parasite resistance and resource allocation, and three growth proxies) were analyzed. Biological trait values of *N. melanostomus* hardly correlated with the environment, could not explain invasion progress and imply a general low overall importance for invasion success. Instead, alternative individual life‐history trajectories appear to determine invasion success. This is in line with up to 15% of all specimens having outlying biological trait values of potential adaptive value, suggesting a considerable importance of adaptive trait variation among single individuals for the whole invasion progress. This “individual trait utility hypothesis” gives an alternative explanation for success of invasive species by single individuals carrying particular traits, and it should be specifically targeted and analyzed at currently invaded sites.

## 1 Introduction

Biological species invasions are a major result of human‐caused global change (Früh, Stoll, & Haase, 2012a) and one of the major threats to biodiversity (Keller, Geist, Jeschke, & Kühn, 2011), especially in freshwater habitats (Geist, 2011). Although invasive alien species (IAS) do not represent a random selection of taxa (Karatayev, Burlakova, Padilla, Mastitsky, & Olenin, 2009), prediction of the invasion progress has remained difficult (Whitney & Gabler, 2008). Numerous hypotheses have been proposed to explain invasion success, for example the *tens rule hypothesis* (Williamson et al., 1986), the hypothesis of *novel weapons* (Callaway & Aschehoug, 2000), of *enemy release* (Colautti, Ricciardi, Grigorovich, & MacIsaac, 2004), and of *invasional meltdown* (Simberloff & Von Holle, 1999). However, a global literature review revealed that several hypotheses have little power to explain and predict biological invasions, that their applicability is sometimes limited, and that hypotheses taking into account the invaded environment have higher empirical support than hypotheses focusing on single species‐specific criteria or single environmental factors (Jeschke et al., 2012). The low applicability of generalizing hypotheses may indicate that invasion success is multifactorial and that it rather depends on a combination of site‐ and habitat‐specific settings. In addition, varying characteristics and traits of single individuals may also be crucial (Nilsson, Brönmark, Hansson, & Chapman, 2014).

Nonindigenous species are simultaneously exposed to several new and multifactorial stressors in the invaded systems (Strayer, Eviner, Jeschke, & Pace, 2006). Local impacts may leave both, individual and site‐specific signatures in the genotype and the phenotype. This study attempts to identify such potential signatures of environmental variables by analyzing biological trait variation of invasive alien *Neogobius melanostomus* (Pallas 1814) (Teleostei: Gobiidae) individuals along a chain of sampling sites along a recently invaded river stretch.

An ongoing invasion along a fluvial ecosystem is a particularly appropriate model system, because species characteristics may vary with particular environmental factors along two dimensions only (Sakai et al., 2001). In such a spatially restricted ecosystem, crucial characteristics for invasion success could be identified along small geographic scales considering environmental factors and time since invasion. Such candidate species characteristics may relate either to life‐history (van Kleunen, Dawson, Schlaepfer, Jeschke, & Fischer, 2010), phenotypic plasticity (Cerwenka, Alibert, Brandner, Geist, & Schliewen, 2014), or to tolerance for anthropogenic disturbance and habitat degradation (Allendorf & Lundquist, 2003). Besides, the genomic constitution (Lambrinos, 2004) appears important, too: Matesanz, Horgan‐Kobelski, and Sultan (2014) showed quantitative variation of genetically determined traits and linked genetic variability with local adaptation and invasion progress. However, the genetic variability of invasive populations often varies substantially and depends on propagule pressure, that is, number of introduced specimens and frequency of introduction events (Wilson, Dormontt, Prentis, Lowe, & Richardson, 2009). Propagule pressure is strongly influenced by factors of passive translocation and of primary dispersal (e.g., transportation vessels), whereas active migration propensity of IAS may particularly influence secondary dispersal (area expansion) and population admixture after introduction.

**Figure 1:**
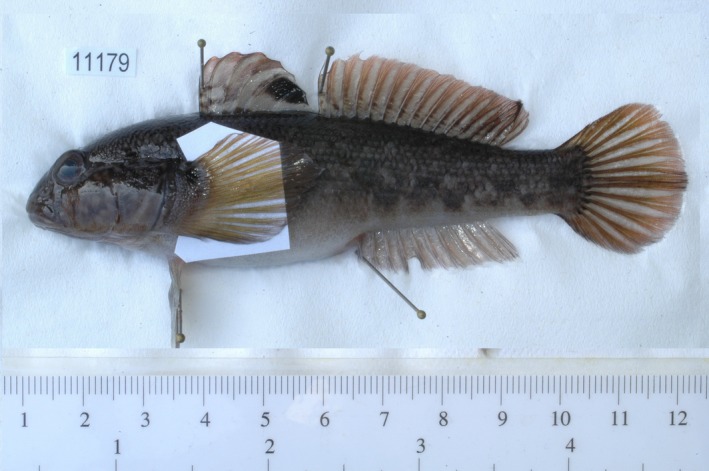
*Neogobius melanostomus* specimen

In recent years, *N. melanostomus* (Figure 1) has become a prime model in invasion biology, because this species has been globally successful in different ecosystems and habitats. It has invaded numerous large waterways such as the Rivers Danube (e.g., Brandner, Cerwenka, Schliewen, & Geist, 2013), Rhine (e.g., Borcherding et al., 2013), Oder (e.g., Schomaker & Wolter, 2014), the Baltic Sea (Kotta, Nurkse, Puntila, & Ojaveer, 2016), and the North American Laurentian Great Lakes (e.g., Corkum, Sapota, & Skora, 2004), making it one of the most prominent IAS worldwide (Lowe, Browne, Boudjelas, & De Porter, 2000). In the upper Danube River, this species was first detected in the year 2004 (Paintner & Seifert, 2006), and one decade later, it has become the most abundant fish species in nearshore habitats (Brandner, Pander, Mueller, Cerwenka, & Geist, 2013). Recent studies have identified several characters of this species, potentially contributing to its success: a predacious generalistic feeding strategy (Brandner, Auerswald, Cerwenka, Schliewen, & Geist, 2013), phenotypic plasticity (Cerwenka, Alibert, et al., 2014), as well as rapid and localized genetic population differentiation (Cerwenka, Alibert, et al., 2014). *Neogobius melanostomus* shows rapid population expansions (Brandner, Cerwenka, et al., 2013; Kornis, Mercado‐Silva, & vander Zanden, 2012), although most individuals appear to cover only limited migration distances, sometimes even restricted to single river sides (Brandner, Auerswald, Schäufele, Cerwenka, & Geist, 2015). However, individual migration propensity appears to be crucial for range expansion and dispersal (Bronnenhuber, Dufour, Higgs, & Heath, 2011), at least in this species.

After range expansion, additional traits may become important for successful establishment of novel populations. Particularly for this phase, which undoubtedly is crucial for long‐term success, the importance of environmental factors is poorly understood and still understudied.

If success of IAS is indeed controlled by specific environmental variables (van Kleunen et al., 2010), specimens’ biological trait values should vary along an environmentally heterogeneous fluvial gradient, but should vary little within single sites within this gradient. Alternatively, success of IAS may be determined by single individuals (see Nilsson et al., 2014). Such “high‐performance” individuals would be characterized by biological trait values that strongly deviate from population average and individuals would have outlying biological trait values, not related to particular environmental factors (Biro & Stamps, 2008; Cote, Clobert, Brodin, Fogarty, & Sih, 2010).

To explore these alternatives, we addressed the following two research questions: (1) Are there significant differences in variability of biological trait values, particularly in outlier frequency, of *N. melanostomus* between sampling localities, and (2) are these differences of biological trait variation correlated with specific variables? If significantly more individuals with biological trait outlier values were detected at expanding frontend populations and if variation of biological traits was not correlated with environmental variation, this would support the hypothesis that high‐performance individuals contributed more to invasion success than environmental variation.

## 2 Material and Methods

Environmental data and *N. melanostomus* specimens (Figure 1) were collected at 158 sampling sites along a 200 km stretch of the upper Danube River from March 2010 until October 2011. Subsequently, seven biological traits (nutrition and energetic status, parasite resistance, resource allocation and three growth proxies) of 653 *N. melanostomus* specimens were measured and averaged per sampling site. Finally, potential associations and correlations with environmental variables were investigated.

### 2.1 Field sampling

**Figure 2:**
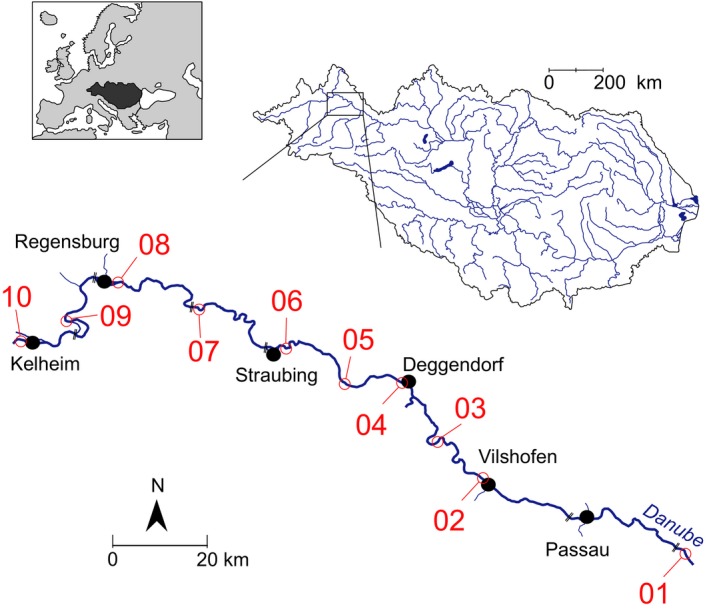
Distribution of ten sampling areas, comprising 158 sampling sites at both river sides with two mesohabitat structures (rip‐rap and gravel), within a 200 river‐km stretch along the Upper Danube River

Sampling covered two entire growth seasons of *N. melanostomus* (see Brandner, Auerswald, et al., 2013; Brandner, Cerwenka, et al., 2013; Cerwenka, Brandner, Geist, & Schliewen, 2014; Cerwenka, Alibert, et al., 2014) with a maximal time period between samplings of 1 month. Sampling areas (Figure 2, Table 1) comprised both river sides and two mesohabitat structures (artificial stone blocks and natural gravel banks).

**Table 1:** Ten representatively distributed sampling areas along the upper Danube River sorted from upstream to downstream. Upper and lower boundaries of sampling areas were delineated by river kilometers (rkm) and GPS coordinates

Sampling area		Lower boundary		Upper boundary	
No.	Name	rkm	GPS	rkm	GPS
10	Kelheim	2,409	E 11°56′27″ N 48°54′29″	2,418	E 11°50′12″ N 48°54′01″
09	Bad Abbach	2,393	E 12°00′13″ N 48°57′57″	2,400	E 12°02′05″ N 48°56′03″
08	Regensburg	2,373	E 12°10′41″ N 49°00′34″	2,377	E 12°08′29″ N 49°01′22″
07	Geisling	2,350	E 12°23′37″ N 48°58′51″	2,354	E 12°21′02″ N 48°58′36″
06	Straubing	2,309	E 12°42′26″ N 48°53′34″	2,317	E 12°36′56″ N 48°53′49″
05	Mariaposching	2,292	E 12°52′12″ N 48°50′28″	2,298	E 12°47′46″ N 48°49′33″
04	Deggendorf	2,280	E 12°59′50″ N 48°47′31″	2,289	E 12°54′26″ N 48°50′40″
03	Aichet	2,267	E 13°03′08″ N 48°43′37″	2,273	E 13°02′15″ N 48°44′32″
02	Vilshofen	2,250	E 13°10′44″ N 48°38′24″	2,259	E 13°05′41″ N 48°41′02″
01	Engelhartszell	2,196	E 13°46′29″ N 48°28′32″	2,202	E 13°43′21″ N 48°30′48″

At each sampling site, electrofishing (ELT62‐IID; Grassl GmbH, Berchtesgaden, Germany) of 30 PAS (point abundance sampling) points was conducted at ~60 cm water depth with a distance of ten meters between points following a standardized methodology as described in Brandner, Cerwenka, et al. (2013). Environmental variables were recorded in two different hierarchical levels: PAS point (at each PAS point: *n* = 3,854) and site specifically (at PAS points 5, 15 and 25: *n* = 474). PAS point‐specific environmental variables were as follows:

1. *habitat quality*, that is, estimations of the granulometric substrate composition according to Jowett and Richardson (1990) (bedrock [>400 mm], boulder [256–400 mm], cobble [64–256 mm], gravel [2–64 mm], sand [0.06–2 mm], and silt [<0.06 mm]),
2. *fish community*, that is, *nongobiid* and *gobiid* fish densities measured as catch‐per‐unit‐effort (CPUE),
3. *water current*, measured in m/s, 10 cm above the bottom (Höntzsch Instruments, Waiblingen, Germany)

Site‐specific environmental variables were as follows:

1. benthic *macroinvertebrate community*, following Brandner, Auerswald, et al. (2013), that is, preserved macroinvertebrate samples were determined to the lowest possible taxonomic unit using a stereo microscope. Estimated volumetric proportions were summarized to ten categories: (1) Amphipoda, (2) Gastropoda, (3) Diptera, (4) Copepoda, Cladocera and Ostracoda, (5) EPT, that is, the three insect orders Ephemeroptera, Plecoptera and Trichoptera, (6) Isopoda, (7) Bivalvia, (8) Bryozoa, Porifera and Hydrozoa, (9) Oligochaeta, and (10) rest, that is, all other organisms.
2. *water quality*, that is, turbidity [NTU], temperature [°C], pH, O_2_ [mg/L], electrical conductivity [uS/cm].

For a site‐specific characterization of the goby populations, the following variables were measured:

1. *total length* (*L*_*T*_) to the nearest mm,
2. *wet mass* to the nearest 0.2 g and
3. *sex* which was determined by the morphology of the urogenital papilla (Brandner, Auerswald, et al., 2013).

### 2.2 Biological traits

Adult and mature specimens with a proximal *L*_*T*_ of 8–12 cm (range = 2.9–15.2 cm, mean = 9.6 cm, *SD* = 1.5 cm) were anaesthetized and killed with an overdose of MS‐222, immediately frozen on dry ice, and stored in the Ichthyology department of the Bavarian State Collection Munich (ZSM). For each specimen, seven biological traits were quantified (allocation of traits was independent of size and sex; Table 2):

**Table 2:** Investigated biological traits of 653 *Neogobius melanostomus* specimens, their full names, and their abbreviations, units, justification for the analysis and the corresponding reference(s)

Biological trait (abbreviation)	Unit	Justification	Method described in
Condition factor (CF)	[g/cm^3^]	Indicator for the nutritional state and the feeding activity, as specimens were size selected and seasonal as well as sex‐related feeding differences are small.	Brandner, Auerswald, et al. (2013)
Number of subadult acanthocephalans (parasites)		Indicator for parasite resistance trade‐offs and costs of parasite defense. Acanthocephalan larvae of the genus *Pomphorhynchus* spp. attached to kidney, liver, gonads, spleen, and surface of the intestinal tract were counted.	Simková et al. (2008), Ondračková et al., (2014) and Brandner, Auerswald, et al. (2013)
Lipid content (*lipids*)		Lipids were used to quantify the ecological differentiation. The isotopic C/N ratio was calculated from muscle tissues.	Post et al. (2007) and Brandner, Auerswald, et al. (2013)
Hepato‐somatic index (*HSI*)		Indicator for the energetic status and general condition.	Dempster et al. (2011) and Azour et al. (2015)
Growth rate (annuli)	[μm]	Distance between the first and the second annulus as a correlate for different resource allocation.	Heino and Kaitala (1999) and Martin (2012)
Growth rate (# *circuli*)		Number of circuli between the first and the second annulus, as a correlate for different resource allocation.	Heino and Kaitala (1999) and Martin (2012)
Growth heterogeneity (*var circuli*).		Variance in circuli spacing between the first and the second annulus as a correlate for different resource allocation.	Heino and Kaitala (1999) and Martin (2012)

1. *Condition factor* (CF [g/cm^3^]), calculated following Brandner, Auerswald, et al. (2013): $\text{CF}=100\times \left(M_{f}-M_{g}\right)/L_{T}^{3}$

with *M*_f_ = fish body mass [g], *M*_g_ = gut content mass [g].

- Number of subadult acanthocephalans (*parasites*), counted using a stereo‐microscope following Ondračková et al. (2014).
- Lipid content (lipids), calculated for muscle tissues following Post et al. (2007).
- Hepato‐somatic index (HSI), calculated following Brandner, Auerswald, et al. (2013): $\text{HSI}=100\times \left(\frac{M_{\mathrm{river}}}{M_{S}}\right)$

with *M*_S_ = *M*_f_ − (*M*_gonads_ +* M*_g_).

- Three individual growth proxies (*growth*). For each specimen, a fish scale with clearly visible and intact circuli (growth‐associated ridges on scales) was selected. Scales were cleaned by panning in chlorine cleaner (DanKlorix, Colgate‐Palmolive GmbH, Hamburg, Germany), washed with tap water, and photographed using a camera (DP 72, Olympus Corporation, Hamburg, Germany) installed on a binocular (Olympus SZX 10, Olympus Corporation). A benchmark was placed on each photograph using the software “Cell” (Olympus Corporation). All measurements were conducted along the longest radius of each scale between the first and the second annulus with Photoshop cs5 extended version 12.0.04 x64 (Adobe Systems, San Jose, CA, USA). Scale readings (Martin, 2012) were used to quantify growth rate (distance between *annuli* [μm] and number of circuli [# *circuli*]) and growth heterogeneity (variance in circuli spacing [*var circuli*]).

### 2.3 Data analyses

Environmental data were reduced by principal component analyses (PCA) and only Principal components (PCs) exceeding the Broken Stick distribution (Jackson, 1993) were used. Subsequently, following Früh et al. (2012a), Früh, Stoll, and Haase (2012b), site‐specific environmental data were utilized to group *N. melanostomus* specimens, that is, those caught at sites characterized by higher or lower environmental PC values compared to the median PCs of the corresponding variable at all sites. Finally, biological traits of *N. melanostomus* groups were statistically compared using pairwise Mann–Whitney *U* tests.

Infrequently occurring variables may strongly influence ordination (ter Braak, 2002). Thus, all pairwise Spearman correlation tests were conducted twice, using untransformed and transformed data: CF and species abundance data (*fish* and *macroinvertebrate* communities) were log(x + 0.5)‐transformed, *water quality* measurements and all other biological traits were log(x)‐transformed, and *habitat quality* measurements were converted using arcsine(x).

Canonical correspondence analyses (CCA) combine regression and ordination methods (ter Braak, 1986) and were applied using the software CANOCO 4.51 (ter Braak, 2002). For CCA only, missing environmental data (*habitat quality*:* n* = 36, 22%; *water quality*:* n* = 17, 11%; *current*:* n* = 41, 26%; *macroinvertebrates*:* n* = 14, 9%; *fish communities*:* n* = 26, 16%) and missing biological traits (CF: *n* = 1, <1%; HSI: *n* = 321, 51%; parasites: *n* = 0, 0%; scale measurements: *n* = 225, 34%) were complemented by hypothetical plausible values. Estimations were conducted by multiple imputations under a multivariate normal model using the software *mice* (multivariate imputation by chained equations) with default settings (van Buuren & Groothuis‐Oudshoorn, 2011) and the predictive mean matching method (Little, 1988). Effects of potential confounding variables were minimized by treating them as covariables, that is (1) *season*, (2) *year,* and (3) *area*. To assess the significance of each environmental variable, the automatic forward selection in CANOCO was used with 9,999 random permutations and Monte Carlo permutation tests.

Variation of each of the seven biological traits (CF: *n* = 97, HSI: *n* = 82, parasites: *n* = 146, scale measurements: *n* = 139) was graphically displayed using boxplots. Therefore, trait distributions were classified for the variables (1) *area*, (2) *river*‐*side*, (3) *habitat quality*, (4) *season*, (5) *year*, and (6) all sites together and analyzed independently (n_boxplots_ = 49). Further, specimens that deviated more than 1.5 times from the interquartile range of the all sites together dataset were identified as outliers in PAST 3.06 (Hammer, Harper, & Ryan, 2001). To validate the number of specimens carrying outlying trait values, 1,000 potential trait allocations were estimated at each sampling site, following Dawson (2011). Therefore, the extreme values of each trait in the all sites together real‐world dataset were used as cutoffs. Subsequently, the mean number of extreme outliers of both datasets (real‐world vs. estimated) were compared using a parametric t‐test. The number of specimens carrying outlying biological traits at each river area was compared using pairwise Bonferroni corrected Mann–Whitney *U* tests.

## 3 Results

Only few significant (*p* < .05) pairwise correlations of biological traits with ecological variables and covariables were detected; that is, only few biological traits were linked to feeding resources, anthropogenic stressors, water temperature, migration barriers, and to covariables (*area*,* season,* and *year*). Trait distribution was mostly independent of sampling area and locality, and associations of biological trait values with environmental variables were weak in the CCA (all eigenvalues < 0.016). Still, a considerable number of 99 specimens had outlying biological trait values, that is, traits of 15.2% of all specimens distinctively deviated from all other individuals. Thereby, the number of specimens having such outlying biological traits significantly deviated from hypothetical mean, for all traits.

### 3.1 Environmental factors and biological trait values

Environmental factors varied considerably along the upper Danube River. They were simplified by five PCAs and summarized by PCs exceeding the Broken Stick distribution. The first PC of *habitat quality* accounted for 66.3% of the variance with substrate having the highest contribution [bedrock (loading = 0.77), sand (loading = −0.53), and gravel (loading = −0.32)]. PC 1 of the *macroinvertebrate* species community accounted for 99.4% of the variance, and amphipods had the highest loading (0.86). The *gobiid fish* community was strongly dominated by *N. melanostomus* (PC1: 99.8% of the variance, loading = 1.00). PC 1 of *water quality* accounted for 67.3% of the variance and the factor with the highest loading was *water current* (0.98), whereas PC 2 accounted for 24.9% and *turbidity* showed the highest loading (0.97). The *nongobiid fish* community was mirrored by the first two PCs (PC 1 = 66.4% and PC 2 = 21.6% of the variance). The nongobiid fish species contributing most to PC 1 was *Alburnus alburnus* (loading = 1.00), and to PC 2, it was *Leuciscus idus* (loading = 0.98). Results using normalized environmental variables (not shown) were highly similar to the results computed without normalization.

*Neogobius melanostomus* was found at all sites. Specimens inhabiting artificial stone blocks had significantly higher condition factors (*CFs*) compared to those from more natural sites (Mann–Whitney *U*,* p *<* *.001). In addition, the *CF* was significantly higher at sites with high *macroinvertebrate* species community PC values (Mann–Whitney *U*,* p *<* *.001), which in turn was associated with *habitat quality*:* Dikerogammarus* spp. were 3.8 times more abundant at sites altered by artificial stone blocks than at more natural gravel banks.

Further, *CF* and *lipids* were significantly associated with *water quality* (PC 2: 96.7% of the variance in the dataset). *CF* was significantly higher at sites characterized by high water turbidity (Mann–Whitney *U*,* p* < .05), whereas *lipids* were significantly (Mann–Whitney *U*,* p *<* *.001) increased when *turbidity* was lower. In addition, elevated *lipids* covaried with (1) higher abundance and density of gobies, (2) a higher temperature regime (i.e., in the late season of both years), and (3) longer time since invasion. Likewise, *parasites* were significantly higher in 2011 than in 2010 (Mann–Whitney *U*,* p *<* *.05), but their abundance was significantly lower at the lowermost part of the sampled river stretch.

**Figure 3:**
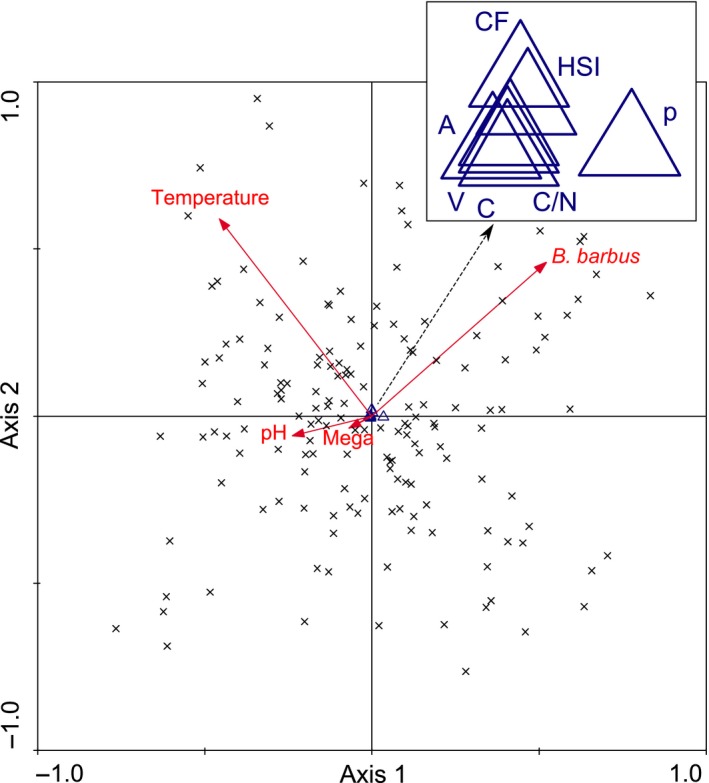
Partial canonical correspondence analysis (CCA) triplot diagram of *Neogobius melanostomus* (*n* = 653) based on seven biological traits, 24 environmental variables, and five covariables at 158 sites along the upper Danube River. Four environmental variables were taken into account using the forward selection (pH, Megalithal (Mega), temperature and number of *Barbus barbus*). The eigenvalues of axis 1 and axis 2 were 0.02 and 0.00, respectively. Sites are labeled with black crosses, averages of biological traits with blue diamonds (p = parasites, HIS = hepato‐somatic index, C/N = lipids, A = annuli, V = variance circuli, C = number of circuli) and environmental variables by red arrows

Simultaneously, *fish*‐species composition differed with *habitat quality*:* N. melanostomus* and *Squalius cephalus* were the species contributing highest to the first PC at artificial habitat structures, whereas *A. alburnus* was 3.6 times more abundant at natural gravel than at artificial sites. The abundance of *Barbus barbus* was negatively correlated with the abundance of *N. melanostomus* (data not shown), with *water temperature* (in our CCA), and it decreased with time since invasion of *N. melanostomus* (Figure 3; Tables 3 and 4).

**Table 3:** Environmental variables and biological traits of *Neogobius melanostomus* (*n* = 653) at 158 sampling sites along the upper Danube River. For environmental variables, all significant PCs, their variance, and the variable with the highest loading are indicated (variable 1). Biological trait allocation in dependence of the environment was compared by subdividing sampling sites into sites with *N. melanostomus* having lower and higher biological trait values than indicated by the median value (pooled for all sites; CF = condition factor, parasites = number of acanthocephalan parasites, lipids = isotopic ratio, HSI = hepato‐somatic index, annuli = distance between first and second annulus, # Cir = number of circuli between first and second annulus, VarCir = variance of circuli distances between circuli of first and second annulus). The number of sampling sites having lower and higher environmental PC values is denoted for each biological trait and significant differences are indicated by stars (***p < .001, **p < .01, *p < .05)

Environmental variable					Biological trait						
Dataset	PC	Variance [%]	Variable 1	Loading variable 1	CF	Parasites	Lipids	HSI	Annuli	# Cir	VarCir
Habitat quality	1	66.3	Bedrock	0.77	35/56***	55/56	55/56	29/33	50/55	50/55	50/55
Habitat quality	1	66.3	Sand	0.53	35/56***	55/56	55/56	29/33	50/55	50/55	50/55
Habitat quality	1	66.3	Gravel	−0.32	35/56***	55/56	55/56	29/33	50/55	50/55	50/55
Water quality	1	67.3	Current	0.98	49/23	53/37	53/37	28/16	52/32	52/32	52/32
Water quality	2	24.9	Turbidity	0.97	47/25*	53/37	53/37***	25/19	47/37	47/37	47/37
Fishes	1	66.4	*A. alburnus*	1.00	47/50	63/58	63/58	28/35	58/56	58/56	58/56
Fishes	2	21.6	*L. idus*	0.98	36/61***	59/62	59/62	31/32	53/61	53/61	53/61
Gobies	1	99.8	*N. melanostomus*	1.00	31/66**	55/66	55/66	29/34	48/66	48/66	48/66
*macroinvertebrates*	1	99.4	Amphipoda	0.86	40/53***	67/67	67/67	39/37	61/66	61/66	61/66

**Table 4:** Summary statistics of canonical correspondence analysis of seven biological traits of *Neogobius melanostomus* along the upper Danube River

Axes	Values of ordination axis			
	1	2	3	4
Eigenvalues	0.016	0.015	0	0
Biological trait environment Correlations	0.745	0.735	0.566	0.494
Cumulative variance of biological traits [%]	27.7	53.6	53.9	54.2
Cumulative % biological trait environment relation [%]	51.2	98.9	99.5	99.9

Summary statistics of canonical correspondence analysis (CCA): Biological trait values of *N. melanostomus* (*n* = 653) and environmental variables along 158 sites of the upper Danube River. Low eigenvalues indicate no apparent structure.

### 3.2 Individuals and biological trait variation

The number of individuals having outlying biological trait values sensu Tukey (1977) deviated significantly from the hypothetical mean of 1000 trait estimations: Significantly more *N. melanostomus* specimen had outlying high number of *parasites*, a higher *lipid* content, *CF*, var *circuli*,* HIS*; significantly fewer specimens featured significantly increased distances between *annuli* and high #*circuli* (all: t‐test *p *<* *.001). Individuals having outlying biological trait values were significantly more often identified at the uppermost site (i.e., area 10) as compared to the five other river areas (i.e., area 1, 5, 6, 7, 8) (Bonferroni corrected Kruskal–Wallis tests, all: *p *<* *.01). In contrast, no other out of the 55 pairwise biological trait variation comparisons at all river areas was significant (Bonferroni corrected Kruskal–Wallis tests, all: *p *>* *.3). There, considerably more specimens had outlying high values of parasites (counts in 2011, and in the late season of both years). In addition, substantially more specimens showed outlying high lipid content values in 2010, as well as in the early season. In total, 15.2% of all specimens were characterized by outlier values: 55 individuals (8.4%) exhibited an elevated parasitic load, 43 specimens (7.0%) showed increased lipid content, 32 specimens (4.9%) exhibited outlier condition factor values (*CF*: decreased: 5, increased: 11), 14 specimens (3.3%) had an increased variability of growth (var *circuli*), and three specimens (0.9%) revealed an elevated *HSI*. For the other two growth‐related biological traits (*annuli* and # *circuli*), no outlier specimens were detected.

## 4 Discussion

Invasion progress is known to be triggered by both, species‐specific traits (Colautti et al., 2014; Nathan et al., 2008) and environmental factors (van Kleunen et al., 2010; Urban, Phillips, Skelly, & Shine, 2008). Our study supports an additional hypothesis, that is, that the contribution of single individuals is of particular importance (see also: Chapple, Simmonds, & Wong, 2012), especially shortly after first inoculation.

Predictions of invasion success or failure remain difficult, yet would be very useful in terms of deriving management implications: Several studies revealed environmental factors to be crucial for invasion success in invasive alien plants (Kempel, Chrobock, Fischer, Rohr, & van Kleunen, 2013), amphibians (Lindström, Brown, Sisson, Phillips, & Shine, 2013) and in several aquatic species (Urban et al., 2008; Gallardo & Aldridge, 2013; Kornis et al. 2013). Kotta et al. (2016) inferred abiotic hydrological factors in combination with proximity to ports as the most important factors in promoting the spread of invasive alien *N. melanostomus*. In contrast, environmental settings were of inferior importance in our study and we detected only minor or no correlation of biological trait values with environmental factors. Similarly, Konečná, Janáč, Roche, and Jurajda (2016) could not identify any environmental factor correlating with goby population characteristics, either. These authors observed reduced fecundity values in *N. melanostomus* and also in *P. kessleri* and *Neogobius fluviatilis* in invaded habitats and suggested altered environmental settings as a possible explanation. However, reproductive traits may depend on single individuals (e.g., characterized by high fecundity) which may contribute particularly strongly to species invasion progress shortly after inoculation. Thus, environmental conditions in combination with species‐ or individual‐specific (reproductive) biological traits may indeed constrain inoculation and establishment of nonindigenous species (van Kleunen et al., 2010; Matesanz et al., 2014), but after successful inoculation, the environment seems to be of inferior importance, as exemplified in our case.

The Danube River has been heavily altered in both, morphology and temperature. Früh et al. (2012a,b) showed that anthropogenic caused environmental degradation correlates with high abundance of IAS. A potential explanation may be a generally higher tolerance of invaders against stressors (Früh et al., 2012a,b; *N. melanostomus*: Ondračková et al., 2014). However, such anthropogenic alterations, for example, migration barriers, can limit species movement and admixture of genetically differentiated populations and may thus differentially change species fitness (Crispo, Moore, Lee‐Yaw, Gray, & Haller, 2011). In *N. melanostomus,* migration barriers may be important for rapid differentiation (Cerwenka, Alibert, et al., 2014; Cerwenka, Brandner, et al., 2014). However, no coherence of biological trait variation and genetic and morphometric population structure was detected in the present study.

Temperature regimes of the upper Danube River increased almost 2°C over the last 100 years (Bloesch, 2011). In this study, water temperature was slightly but significantly correlated with biological trait values of *N. melanostomus*. Interpretation of this result is not straightforward, because (1) the temperature regime considerably changed during the sampling period, (2) the catchability of *N. melanostomus* changes with water temperature (Brandner, Pander, et al., 2013), and because (3) movement and habitat use of N. melanostomus varies seasonally (Kornis et al., 2012) and thus with water temperature. Similarly, *N. melanostomus* had lower parasite loads at sites characterized by low temperature regimes (e.g., downstream of the influx of the River Inn). As metabolic activity in poikilothermic organisms decreases with decreasing water temperature, a lower nutritional uptake at lower temperatures could be an explanation. However, differences in specimen size may have biased our results: *N. melanostomus* individuals were significantly smaller at sites below the influx of the River Inn (Mann–Whitney *U*,* p* < .001) and thus the number of acanthocephalan parasites per specimen may have been lower, which is in line with the observation of minor importance of environmental factors on biological trait variation.

### 4.1 Dynamics of biological traits in *Neogobius melanostomus*

About 15% of all analyzed *N. melanostomus* specimens had at least one biological trait value deviating from the average variation. Similarly, a subset of all specimens from a population are thought to contribute disproportionately high to the species expansion in the Gulf of Gdansk, Poland (Thorlacius, Hellström, & Brodin, 2015), and the Great Lake tributaries (Bronnenhuber et al., 2011). Thorlacius et al. (2015) found “bolder” *N. melanostomus* individuals to be more aggressive, more explorative, and being more capable to cover greater migration distances. The authors suggest boldness to be beneficial (providing easier access to new resources), but also to involve a trait‐off in terms of higher costs (risk of predation). However, bold (in our case: large–sized and high conditioned) individuals may even benefit from a lower predation risk at (expanding) fronted populations (Brownscombe & Fox, 2012) which might further support their fast dispersal (Brandner, Cerwenka, et al., 2013; Lindström et al., 2013).

Selection operates predominantly at low levels of the hierarchical organization of species, that is, genes and individuals (Lewontin, 1970). Individuals with outlying biological trait values may indeed contribute disproportionally to population fitness (Coulson et al., 2006) (lower in the case of elevated parasitic load (Milinski, 2009)). Such individuals may not dramatically alter average biological trait variation at the population level *ad hoc*, but they nevertheless may significantly influence invasion progress, because they may act beneficially for all members of a rapidly expanding population by increasing their fitness during a critical phase of the invasion process (Smith & Blumstein, 2008). Genome scans revealed that contemporary site‐specific differentiation of *N. melanostomus* (in the same river stretch) can be correlated with just three out of 189 analyzed AFLP‐loci: 178 individuals (31%) carried the one of those, 81 (14%) another one, and 13 (2%) the third one. In the context of the present study, it is noteworthy that three specimens (0.5%) carried all three alleles and that each of these three specimens originated from the expanding frontend population (Cerwenka, Brandner, et al., 2014). Interestingly, the occurrence of all three individuals was not independent from area and time since invasion (χ^2^‐test, *p* = 1). These population genomic results in combination with the results presented herein indicate that single individuals with an exceptionally high fitness may indeed have an effect on invasiveness of a whole population. In this context, Brandner, Cerwenka, et al. (2013) already pointed to a contribution to invasion success of individuals being comparatively large and having a high condition, that is, possibly being bolder.

Surprisingly, and in contrast to Cerwenka, Alibert, et al. (2014), Cerwenka, Brandner, et al. (2014) and Brandner, Cerwenka, et al. (2013), the present study did not find strong trait distribution differences between recently invaded and longer established sites. A possible explanation for this paradox may lie in fast personality‐dependent dispersal (Thorlacius et al., 2015), because high migration rates may rapidly level out small scale trait distribution differences even across large distances (Brandner, Auerswald, et al., 2013; Brandner et al., 2015). In addition, multiple inoculations may have the same leveling effect (Colautti et al., 2004), possibly by increasing the likelihood of introducing locally fit (i.e., well adapted) individuals.

In conclusion, our study demonstrates that biological traits on the level of single individuals appear to determine the success of invasive alien gobies. This “individual trait utility hypothesis” should be specifically targeted and tested in future studies of nonindigenous and native expanding populations.

## Conflict of Interest

The authors declare that they have no conflict of interest.
